# A Methodology for Porcine Circovirus 2 (PCV-2) Quantification Based on Gold Nanoparticles

**DOI:** 10.3390/ma13051087

**Published:** 2020-02-29

**Authors:** Caroline R. Basso, Taís F. Cruz, Bruna L. Silva, Valber A. Pedrosa, João P. Araújo Junior

**Affiliations:** 1 Institute of Biotechnology, UNESP- Botucatu, SP 18607-440, Brazil; tfcruz@yahoo.com.br (T.F.C.); brunaslindolfo@gmail.com (B.L.S.); joao.pessoa@unesp.br (J.P.A.J.); 2 Department of Microbiology and Immunology, Institute of Bioscience, UNESP-Botucatu, SP 18618-000, Brazil; 3 Department of Chemistry and Biochemistry, Institute of Bioscience, UNESP-Botucatu SP 18618-000, Brazil

**Keywords:** biosensor, gold nanoparticles, porcine circovirus 2

## Abstract

The aim of the current study is to introduce a methodology aimed at producing a biosensor that uses gold nanoparticles (AuNPs) to detect porcine circovirus 2 (PCV-2). This biosensor was based on AuNPs, which were modified with self-assembled monolayers (SAMs) and antibodies. The AuNPs’ surface and virus modification process applied to enable antibody binding was accompanied by localized surface plasmon resonance (LSPR), surface plasmon resonance (SPR), transmission electron microscopy (TEM), and energy-dispersive X-ray spectroscopy (EDX). Virus quantification was possible by the light absorption difference in the spectrum at concentrations of 10^5^, 10^6^, 10^7^, 10^8^, and 10^9^ DNA copies/mL PCV-2 in relation to quantitative PCR (qPCR), with an R^2^ value >0.98. The visualization of colorimetric changes in the different PCV-2 concentrations was possible without the use of equipment. The biosensor production methodology presented reproducibility and specificity, as well as easy synthesis and low cost. An enhanced version of it may be used in the future to replace traditional tests such as PCR.

## 1. Introduction

Porcine circovirus diseases (PCVD) is one of the most important diseases affecting domestic swine production; it is caused by porcine circovirus 2 (PCV-2) belonging to the family *Circoviridae*, genus *Circovirus*, which comprises three endemic types, PCV-1, PCV-2, and PCV-3 [1]. PCV-2 is the most relevant pathogen affecting pigs; it accounts for major damages to the economy, since the high mortality rate resulting from it causes irreparable damage losses to farmers [2].

The PCV-2 has a single-stranded DNA genome, with 1760 bases, wrapped by an icosaedric capsid [3]. This virus, which is approximately 17 nm diameter, is the agent responsible for several pig syndromes [4]. Phylogenetic analyses subdivided PCV-2 into five genotypes: PCV-2a, PCV-2b, PCV-2c, PCV-2d, and PCV-2e. Based on epidemiological studies, PCV-2b was the most prevalent after PCV-2a, and today, PCV-2d is widespread in many countries, suggesting a change of genotype from PCV-2b to PCV-2d, which is responsible for most clinical cases of postweaning multisystemic wasting syndrome (PMWS) [5,6]. The emergence of different genotypes derives from nucleotide additions or substitutions. The mutation of one or two amino acids in the cap, particularly in the open reading frame 2 (ORF2) region, may affect the virulence and pathogenicity of PCV-2 [2,6,7]. Moreover, a single animal can be infected with different PCV-2 genotypes, favoring the occurrence of recombination between them [7]. 

PCV-2 can infect wild and domestic pigs presenting different health standards. Viral transmissions result from direct contact with infected animals and through oral, nasal, oropharyngeal, fecal, and urine fluids. There are also reports about transmission through semen and colostrums [8]. PCV-2 infection symptoms comprise reduced weight gain, maternal reproductive failure, congenital tremor, nephropathy, dermatitis, and the most well-known one: PMWS. The main clinical signs of PMWS encompass apathy, anorexia, rapid and progressive weight loss, conjunctivitis, pallor, dyspnea, enlarged lymph nodes, diarrhea, respiratory issues, and jaundice of the skin and mucous membranes [9]. Inadequate herd management and external factors such as temperature and poor air quality can aggravate animals’ clinical symptoms [10]. PMWS-related mortality rate can reach 80% in 5–12 week old piglets, where as it can range from 4% to 30% in piglets up to 24 weeks old. Infected animals present extreme wasting over time, with no possibility of treatment [2]. As PCV-2 is a ubiquitous virus, viral quantification is extremely important, as it is directly related to lesions and zootechnical losses. An increased viral load in animals causes an increased severity of clinical signs. Pigs with severe lesions have rates ranging from 10^9^ to 10^12^ DNA copies/mL; animals with moderate lesions have a rate of 10^7^ DNA copies/mL; mild lesions represent a rate of 10^5^ to 10^6^ DNA copies/mL, and animals with rates below 10^4^ DNA copies/mL do not present clinical signs [11]. PCV-2 infection has a serious economic impact on the worldwide swine industry, having been diagnosed in the five continents, so it is interesting to know which facilities should be included in cleaning and disinfection procedures, as they may represent critical points where PCV-2 tends to accumulate and therefore act as possible sources of infection and/or reinfection [12,13].

PCV-2 diagnosis is initially carried out by veterinarians who analyze animals’ clinical signs and request laboratory tests such as hybridization in situ, quantitative PCR (qPCR), PCR, immunoperoxidase, and immunofluorescence in order to investigate the presence of the antigen or nucleic acid of PCV-2. However, both techniques have some limitations that can influence the outcome, such as expansive sample analysis and the need for specialized professionals and specific equipment to perform them [1,3,14].

PCV-2 prevention and control must be implemented in farms based on hygiene measures, correct feeding, stress reduction, sick animals’ removal, and herd vaccination, which often takes place within the first four weeks of piglets’ lives. However, the vaccine is not yet 100% effective, because factors such as changing manufacturers, conditioning temperature, and non-vaccination of the whole herd enable PCV-2 to keep on affecting animals [9]. PCV-2 is very resistant to environmental conditions such as acid pH, high temperatures, freezing, ultra-violet light, and available disinfectants [10].

The use of nanomaterials to develop a diagnostic platform has been gaining more and more space in recent years. The use of nanopolymers, nanotubes, and nanoparticles represent a growing biosensors production market. More specifically, gold nanoparticles (AuNPs) provide unique features such as biocompatibility and easy protein functionalization, which make them ideal to produce remote analysis kits [15,16]. AuNPs are ideal materials to produce biosensors, since they have unique optical and electronic properties, which make them suitable for immunoassay applications and to be used as colorimetric kits [17]. Among the advantages of working with AuNPs, one can highlight their easy synthesis, low cost, ease of storage, and long-term solution dispersion. The optical properties of AuNPs allow measuring the oscillation of gold atoms’ conductive electrons by means of a spectrophotometer. The reduced absorbance peak and wavelength shift shown on the UV-Vis graph generated by the spectrophotometer show AuNPs surface modifications and complex formations [18,19,20]. The use of AuNPs allows detecting several pathogens found in samples from different sources such as animals [21], humans [22], environment [23], food [24,25] and cell culture [26], which makes it one of the most used nanomaterials.

The aim of the current study was to introduce a methodology aimed at enabling an easy and simple PCV-2 quantification based on the use of gold nanoparticles. In this biosensor development, storage at room temperature (25°) maintained stability without losing the properties of the reagents and was economical, at a cost of $3 per sample.

## 2. Materials and Methods

### 2.1. Chemicals

Gold (III) chloride trihydrate 99.99% (HAuCl_4_); sodium citrate dehydrate 99%; 11-mercaptoundecanoid acid, 95% (MUA); absolute ethanol 99%; N-(3-imethylaminopropyl) -N′ethylcarbodiimide hydrochloride (EDC); N-hydroxysuccinimide (NHS) 98%; phosphate-buffered saline (PBS); glycine–HCl (pH 3.0), and bovine serum albumin solution (BSA) 98% (1 mg/mL^−1^ in 10 mM PBS, pH 7.4) were purchased at Merck (Darmstadt, Germany). The deionized water used in the current study came from a Millipore unit (Burlington, MA, USA). All working solutions were prepared with analytical grade chemicals.

### 2.2. Antibodies

A female rabbit was immunized with purified PCV-2 at 50 µg/mL (*v*/*v*), based on the protocol described by Johnstone and Thorpe 1996. Rabbit IgG antibody anti-PCV-2 was purified with a 5 mL column HiTrap^®^ Protein G (GE Health-care, Buckinghamshire, UK), according to the manufacturer’s instructions. The purified IgG protein concentration was 8 mg/mL, which was based on the BCA methods, using BSA as protein standards, as described by Smith et al., 1985. (Protocol No. 103/2010-CEUA, FMVZ Animal Use Ethics Committee, São Paulo State University-Botucatu [14].

### 2.3. Samples

Thirty positive serum samples with PCV-2 virus presence confirmed by the qPCR technique (≥10^5^ DNA copies/mL of serum) and 10 negative samples also confirmed by the qPCR technique were used in the current study. All samples were collected from animals bred in farms served by the Molecular Diagnostic Laboratory at São Paulo State University, Botucatu, Brazil.

### 2.4. DNA Extraction for qPCR Analysis

DNA was extracted from 100 µL of a pig serum sample by using the Illustra genomic Prep Mini Spin kit (GE Healthcare, Buckinghamshire, UK), according to the manufacturer’s instructions. The DNA was eluted with 100 mL of elution buffer. Extraction control (nuclease-free water) was included in each extraction procedure.

### 2.5. Instruments

The absorbance and wavelength graph was generated in a Biochrom LiraS11 spectrophotometer (Biochrom Ltd., Cambridge, UK) in a 1 cm glass cell, a 1 nm step; a speed of 500 nm min^−1^, and a wavelength accuracy of 0.5 nm. The binding process observed during the experiment was analyzed in surface plasmon resonance equipment (SPR) (AutoLab Springle^®^, Eco-Chemie, Utrecht, Netherlands). The planar gold sensor SPR discs (17 mm diameter) were purchased from Autolab. All experiments were carried out at 22 °C. 

The size and dispersion of gold nanoparticles at different scales were characterized through transmission electron microscopy (TEM), and energy-dispersive X-ray spectroscopy (EDX) in a JEOL-JEM2100 LaB_6_ 200 KV coupled to an EDX operating system (Jeol, Peabody, MA, USA). The copper grids used in the experiment were covered with carbon film and placed in 10 µL of sample; next, they were left to dry at room temperature for two days prior to analysis.

Viral quantification of samples was performed based on the qPCR technique. It was done by using a 7500 Fast Real Time PCR System, Thermo Fisher Scientific, Foster City, CA, USA.

### 2.6. Synthesis of Gold Nanoparticles and Formation of Self-Assembled Monolayers

The synthesis of gold nanoparticles was based on Basso et al. [18]. An Erlenmeyer glass placed on a magnetic stirrer had 10 mL of HAuCl_4_ solution (1 mM) added, which was stirred at 100 °C to the boiling point. Next, 2 mL of sodium citrate (10 mM) was added to the Erlenmeyer glass. The solution, which was yellow, turned red because the sodium citrate reduced the Au^3+^ complex to Au^0^, which led to the formation of nanoparticles. The excess of citrate anions in the solution enabled the gold metal surface to give a negative charge to each nanoparticle. The AuNPs solution was cooled to room temperature and stored in amber glass in a refrigerator (−2 °C). 

Self-assembled monolayers (SAMs) were formed and worked as anchor molecules to enable antibodies to bind to the AuNPs surface. SAMs provided optimum conformational conditions, as well as stability and an excellent microenvironment for biocatalytic activities, which enables biomolecules to guide nanoparticles without denaturation [27]. Alkanethiols were added to a 11-mercapto undecanoid acid (MUA) solution to enable SAMs production. In order to do so, 100 µL of MUA (0.018 mol L^−1^) was added to 2 mL of AuNPs solution for 40 min at 25 °C, which resulted in irreversible thiol adsorption on the AuNPs surface. Next, 100 µL of the 1:1 solution containing EDC (0.4 mol L^−1^) and NHS (0.1 mol L^−1^) was added and remained there for 40 min to activate terminal carboxylic groups that were capable of creating covalent bonds with antibodies’ amines.

### 2.7. Quantitative PCR (qPCR) for PCV

The forward (5′-GAT GAT CTA CTG AGA CTG TGT GA) and reverse (5′-AGA GCT TCT ACA GCT GGG ACA) primers described by Ladekjaer-Mikkelsen et al. [28] were used in qPCR at 0.2 µM. The qPCR was prepared in the GoTaq^®^ qPCR Master Mix kit (Promega, Madison, WI, USA), according to the manufacturer’s instructions. Reaction conditions were 95 °C/2 min, 40 cycles at 95 °C/15 s and 60 °C/1 min, followed by a melting curve from 95 °C to 60 °C. Extraction and reaction controls (nuclease-free water) were processed with the test samples. The viral load was determined based on absolute quantification. A standardized plasmid was diluted from 10^8^ to 10^5^ copies of DNA/mL in duplicate to produce a standard curve. The standard curve was included in each processed plate. Viral load was expressed as the number of DNA copies/mL.

## 3. Results and Discussion

### 3.1. Biosensor Development 

**Figure 1:**
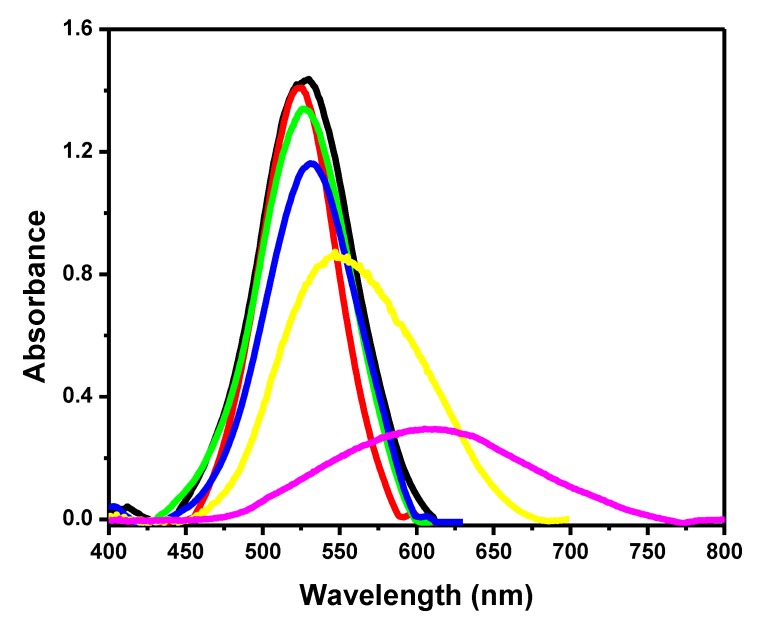
UV-Vis graph showing gold nanoparticles (AuNPs) and modification steps on their surface. AuNPs (black line), mercapto undecanoid acid (MUA, red line), N-(3-imethylaminopropyl)-N′ethylcarbodiimide hydrochloride–N-hydroxysuccinimide (EDC–NHS, green line), antibody 2.5 µg/mL (blue line), 1.8 × 10^7^ DNA copies/mL porcine circovirus 2 (PCV-2) virus (yellow line) and 7.8 × 10^9^ DNA copies/mL PCV-2 (pink line).

All of the AuNPs surface modification stage was monitored through the localized surface plasmon resonance (LSPR) spectroscopy technique; they presented absorbance peak reduction and wavelength shift (Figure 1). Changes in the graph indicated the AuNPs’ dielectric properties.

AuNPs without surface modification showed an absorbance peak of 1.44 at 526 nm (black line); this value is typical of AuNPs, as described in the literature [29]. Thiol bonded to the surface of the AuNPs and formed SAMs after the addition of MUA solution, which decreased the absorbance peak to 1.40 (red line). SAMs’ carboxylic groups were activated by the EDC–NHS solution, and it resulted in an absorbance peak decrease to 1.33 and in a wavelength shift to 529 nm (green line). This experimental step is extremely important because the EDC–NHS solution creates covalent bonds with the antibody amines while keeping nanoparticle–antibody complex stability. The antibody (2.5 µg/mL) addition resulted in a wavelength shift to 532 nm and decreased the absorbance peak to 1.16 (blue line). This change has indicated antibody binding to AuNPs, as reported in the literature [30]. Two pig serum samples testing positive for PCV-2 concentrations were added. The first sample contained 1.8 × 10^7^ DNA copies/mL PCV-2; after their addition, there was an absorbance peak decrease to 0.86 and wavelength shift to 550 nm (yellow line). The second sample plotted on the graph had a PCV-2 concentration of 7.8 × 10^9^ DNA copies/mL, which resulted in a significant absorbance peak decrease to 0.30 and wavelength shift to 610 nm (pink line). The addition of both samples indicated antigen–antibody binding and complex formation. All samples were quantified based on the qPCR technique.

**Figure 2:**
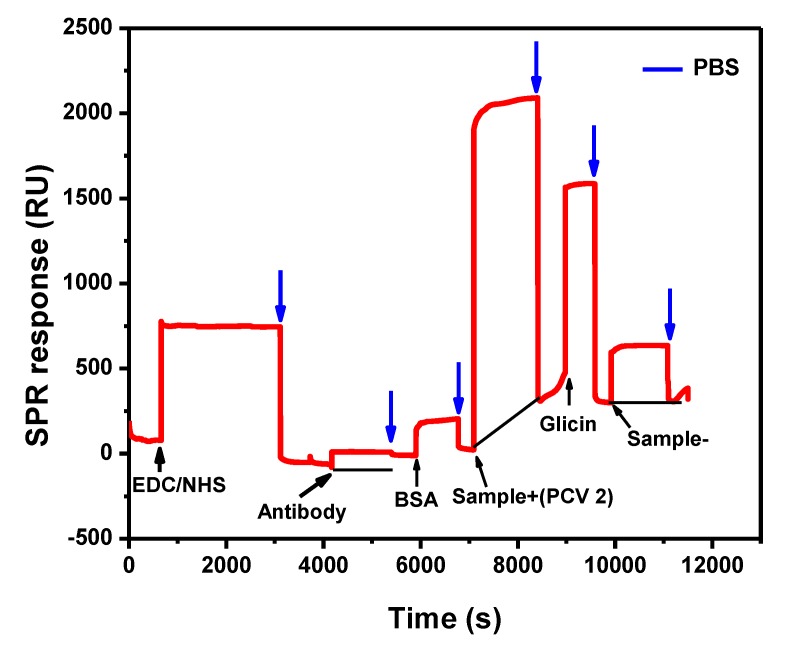
Surface plasmon resonance (SPR) graphic for adsorption and desorption kinetics in each modification. All washing steps of experiment used phosphate-buffered saline (PBS) at pH 7.4, as represented by the blue arrows pointing down (↓).

All modification steps were also followed by surface plasmon resonance (SPR), which enabled adsorption and desorption kinetics in each modification at real time (Figure 2). According to this technique, a gold sensor is used as the basis to immobilize molecules on its surface. The graph has two axes: × and y; the × axis is expressed in seconds and indicates the time of the experiment, while the y axis shows the resonant units (RU), indicating the amount of sample that has been attached to the sensor surface after each washing procedure. According to the equipment’s manufacturer, 1 ng mm^−2^ of material attached to the gold sensor corresponds to 120 millidegrees in the plasmon resonance angle [29].

The gold sensor surface was cleaned with ultrasound detergent for 30 min. Next, it was washed 10 times with deionized water and dried with lens paper. The sensor was inserted into the equipment, and 100 µL of the MUA solution was added to its surface to enable SAMs formation. The solution remained there for 24 h. Then, the sensor surface was washed with pH 7.4 phosphate buffer (PBS) to remove the excess of MUA from it. EDC–NHS solution was added to activate the carboxylic groups and remained there for 30 min before it was washed with PBS. The aliquot of 100 µL of antibody was injected at 2.5 µg/mL and remained there for 30 min. After the washing procedure was over, there was slight drop in the SPR chart line, which indicated a RU of 1235 equivalent to 10.3 of antibodies attached to the sensor. Then, BSA solution (1 mg/mL) was added and used to fill the blanks on the sensor surface in order to avoid false positive connections. Washing with PBS has removed the unbound material. The 50-µL aliquot of PCV-2 sample (7.8 × 10^9^ DNA copies/mL) was added and remained there for 30 min; after the washing procedure was over, there was small drop in the graph in comparison to the virus injection point. This outcome indicated that the virus bond to the antibody immobilized on the sensor surface. Glycine-HCL solution (100 µL) was added and remained there for 15 min, so it was possible going on with the experiment by using a different sample. Glycine–HCL broke the antigen–antibody binding, but it preserved the SAMs/antibody/BSA complex on the sensor surface, which was ready to analyze the second sample. The aliquot of 100 µL of PCV-2 virus negative serum sample subjected to qPCR was added in order to allow analyzing the binding specificity of the immobilized antibody on sensor surface. This sample was used a negative control in the experiment, and it remained on the sensor surface for 30 min. After the washing procedure was over, there was total drop in the graph line in comparison to the negative sample injection point, which indicated antigen–antibody binding specificity. The antibody did not recognize any protein normally present in swine serum that could react with it, such as albumin. After the injection of each solution and their respective incubation time, the sensor surface was washed with PBS buffer to remove the weakly bonded molecules and excess of molecules. All these steps are shown in the graph by the blue arrows pointing downwards.

### 3.2. The Dynamic Range of Signal

**Figure 3:**
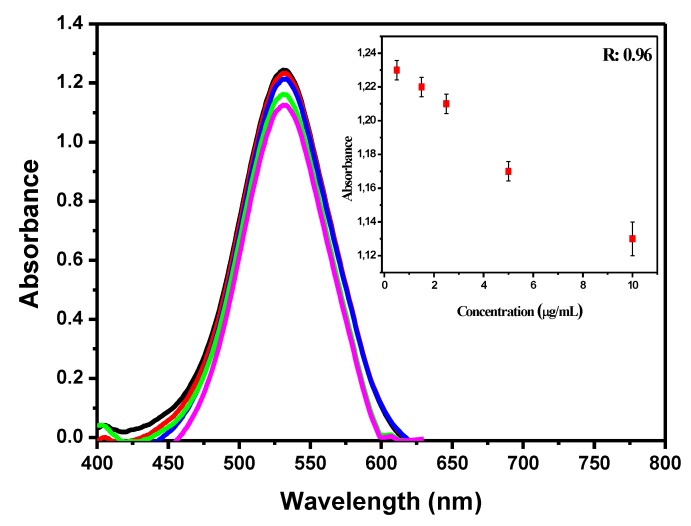
UV-Vis graphic with different antibody concentrations. 0.5 μg/mL (black line), 1.5 μg/mL (red line), 2.5 μg/mL (blue line), 5.0 μg/mL (green line), and 10.0 μg/mL (pink line). Calibration curve with R^2^ 0.96 is inserted in the figure. All dilutions were performed in phosphate buffer (PBS).

Solutions with antibody concentrations of 0.5, 1.5, 2.5, 5.0, and 10.0 µg/mL were tested to find the optimal concentration to work (Figure 3). The solution with 0.5 µg/mL antibody concentration showed an absorbance peak of 1.24 with a wavelength of 532 nm (black line). The second concentration (1.5 µg/mL) showed a small drop in the graph to an absorbance peak of 1.23 at 532 nm (red line). The concentration 2.5 µg/mL showed an absorbance peak of 1.17 at the 532 nm wavelength (green line). The concentration 5.0 μg/mL showed an absorbance peak reduction in comparison to the previous 1.13 at 532 nm (blue line). The last measured antibody concentration (10.0 µg/mL) showed an absorbance peak of 1.10 at 532 nm (pink line). The comparison among drops in all the absorbance peaks allowed seeing that the antibody concentration of 2.5 μg/mL showed the largest difference in absorbance peak in comparison to concentrations lower and higher than it. There was not any wavelength shift in any sample, and the maximum absorbance peak remained at 532 nm. The solution with 2.5 µg/mL antibody concentration was selected to be used in the experiments because of this outcome and to avoid wasting antibodies that were not bond to AuNPs. The calibration curve of the five antibody concentrations (0.5, 1.5, 2.5, 5.0, and 10.0 µg/mL) was inserted in the figure and showed an R^2^ value of 0.96.

This drop in behavior at the absorbance peak in solutions with higher antibody concentrations, as shown in the graph, was also reported in studies by Basso et al. [18] and Wang and Irudayaraj [31]. This response happens because the more modified the AuNPs surface, the harder it is for the laser light of the equipment to interact with its surface.

**Figure 4:**
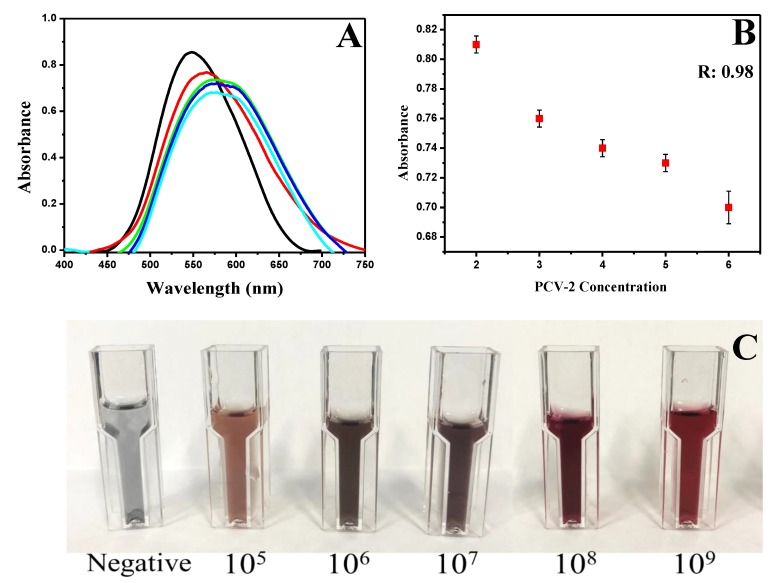
Analysis of different viral loads of PVC-2. (**A**) UV-Vis graphic with different PCV-2 concentrations. 10^5^ (black line), 10^6^ (red line), 10^7^ (green line), 10^8^ (blue line), and 10^9^ (pink line). All values are expressed in DNA copies/mL PCV-2. (**B**) Calibration curve with R^2^: 0.98. All dilutions were performed in PBS. (**C**) Colorimetric change for different PCV-2 concentrations. Negative (gray color), 10^5^ (brown color), 10^6^ (dark gray color), 10^7^ (purplish tone), 10^8^ (wine color), and 10^9^ (red color).

Thirty serological samples collected from different animals with PCV-2 were tested, and a calibration curve was plotted to validate the biosensor functioning (Figure 4). All samples were previously analyzed and quantified based on the qPCR, and they presented concentrations of 10^5^, 10^6^, 10^7^, 10^8^, and 10^9^ DNA copies/mL virus (six samples each) (Figure 4A). One sample with each virus concentration was plotted to make the graphic display easier. The first sample added to the AuNPs/SAMs/antibody complex at a PCV-2 concentration of 8.0 × 10^5^ DNA copies/mL showed an absorbance peak of 0.85 at a wavelength of 547 nm (black line). The red line on the graph showed the concentration of 8.8 × 10^6^ DNA copies/mL with an absorbance peak of 0.75 at 564 nm. The concentration of 1.8 × 10^7^ DNA copies/mL showed an absorbance peak of 0.73 at 574 nm (green line). The blue line showed an absorbance peak of 0.71 at 577 nm for the concentration 1.8 × 10^8^ DNA copies/mL, and the last concentration (7.8 × 10^9^ DNA copies/mL) showed an absorbance peak of 0.68 at wavelength of 580 nm (pink line). Based on this graph analysis, it was possible observing an absorbance peak intensity decrease and wavelength shift in samples with higher PCV-2 concentrations. Similar results were observed by Basso et al. [18] and Wang and Irudayaraj [31], and they can be explained by the difficult interaction between light photons and electrons in the conduction band (free electrons), resulting in a decrease in the absorbance peak and wavelength shift [32]. The calibration curve of the five PCV-2 concentrations (10^5^, 10^6^, 10^7^, 10^8^, and 10^9^ DNA copies/mL virus) showed an R^2^ value of 0.99 (Figure 4B). Variations in viral load concentrations in serum samples showed different colorations that could be observed with the naked eye (Figure 4C). Negative samples without virus presence when added to the AuNPs/SAMs/antibody complex showed a gray color. The concentration of 10^5^ DNA copies/mL when added to the complex showed a brown color; the concentration of 10^6^ DNA copies/mL showed the dark gray coloration; 10^7^ DNA copies/mL showed a purplish tone, 10^8^ DNA copies/mL showed a wine color, and finally, 10^9^ DNA copies/mL concentration showed a color change to red when added to the AuNPs/SAMs/antibody complex. The colorimetric change enables the differentiation of viral loads in animal serum samples without the need for specific equipment and laboratories.

This AuNPs/SAMs/antibody/sample PCV-2 complex was measured over the following three months and showed good biosensor stability.

### 3.3. Biosensor Characterization and Negative Control

**Figure 5:**
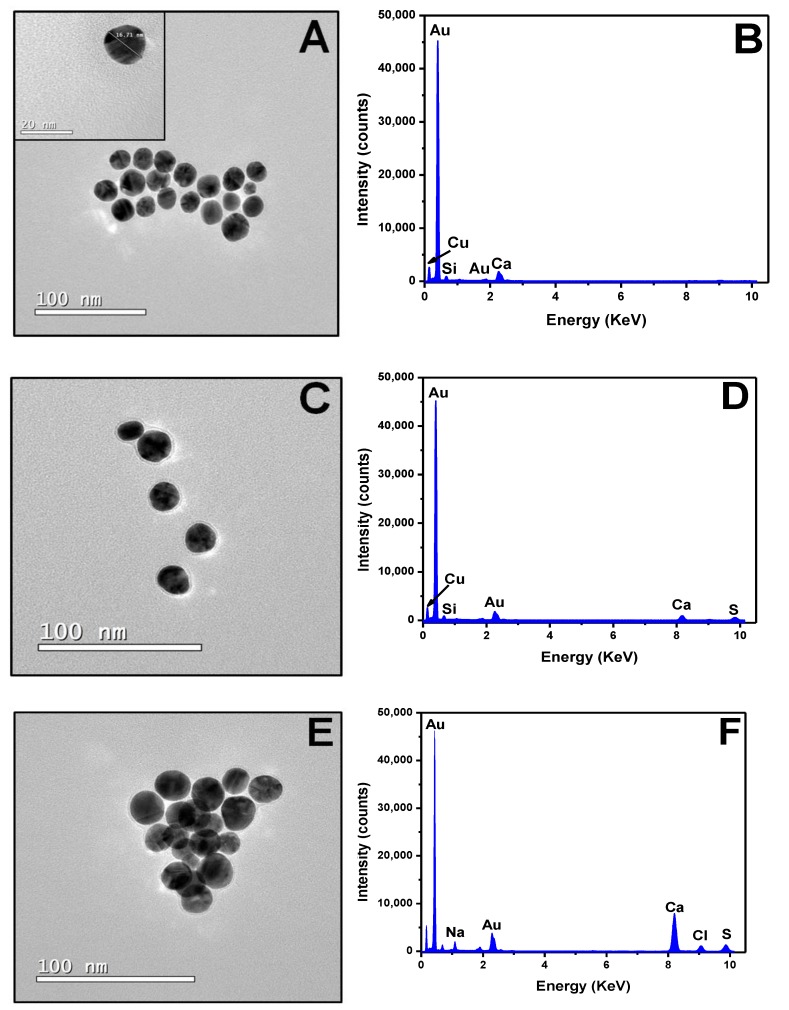

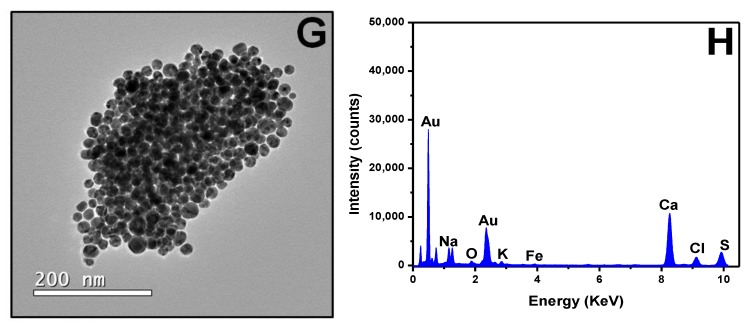
TEM and EDX images generated for all AuNPs surface modification steps. (**A**) AuNPs at a scale of 100 nm. (**B**) AuNPs in the energy-dispersive X-ray spectroscopy (EDX) graphic. (**C**) AuNPs–MUA (mercapto undecanoid acid) and EDC/NHS in a scale of 100 nm. (**D**) AuNPs-MUA and EDC/NHS in EDX graphic. (**E**) AuNPs–MUA–EDC/NHS-antibody (2.5 μg/mL) at a scale of 100 nm. (**F**) AuNPs–MUA–EDC/NHS-antibody (2.5 μg/mL) in the EDX graphic. (**G**) AuNPs–MUA–EDC/NHS–antibody (2.5 μg/mL) sample with 7.8 × 10^9^ DNA copies/mL PCV-2 at a scale of 200 nm. (**H**) AuNPs–MUA–EDC/NHS-antibody (2.5 μg/mL) sample with 7.8 × 10^9^ DNA copies/mL PCV-2 in the EDX graphic.

TEM and EDX images were generated to confirm the UV-Vis graph and view AuNP surface modifications (Figure 5). The first image showed unmodified AuNPs, presenting a diameter of 16.71 nm, on average (Figure 5A). EDX analysis of the AuNPs graph showed a large gold peak for the sample, and copper and carbon peaks for the grid used in the experiment. The small silicon peak was the remnant of the glassware used during AuNPs synthesis (Figure 5B). Figure 5C shows the AuNPs modified with SAMs and activated with EDC–NHS. It is possible observing that thiol formed a shadow around the AuNPs surface, which indicated monolayer formation. EDX analysis showed a new sulfur peak (S) relative to the thiol group (Figure 5D). Similar results were found by Basso et al. [18], who observed SAMs formation in a hybrid nanoparticle composed of iron and gold. Microscopy image 5E showed the AuNPs/SAMs/antibody complex and the bead diameter increased to 30 nm, which indicated antibody presence. The article published by Reth [33] has indicated an antibody size of approximately 12 nm, which was similar to the one found in the current study. EDX analyses (Figure 5F) have shown chlorine and sodium peaks, which indicated the presence of antibodies. These peaks also appeared in analyses performed by Mao et al. [34], who investigated vertically-oriented graphene (VG) sheets labeled with gold nanoparticle (NP)–antibody conjugates. The last grid analyzed in the current study was added with the virus-positive sample to the AuNPs–antibody complex. In this image, it is possible observing a cluster of nanoparticles, as well as the increase in their diameter to 45 nm (Figure 5G). The EDX graph shows new oxygen (O), potassium (K), and iron (Fe) peaks, as well as an increased carbon peak; these elements were found in the serum sample used in the experiment (Figure 5H).

The software in the equipment analyzed AuNPs’ size and dispersion in the grids and provided the herein indicated size (in nm). All the experiments in the current study were performed in triplicate.

### 3.4. Negative control

**Figure 6:**
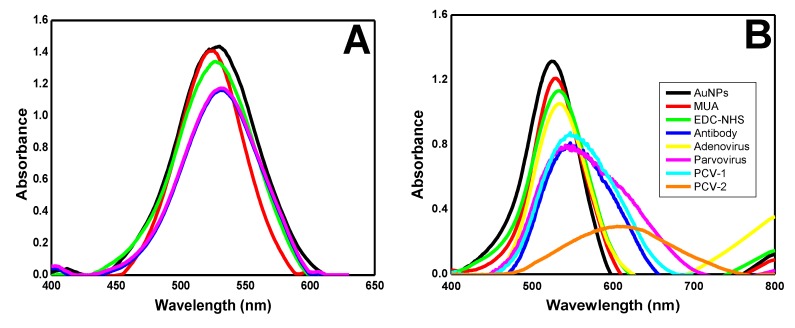
UV-Vis graph showing AuNPs and negative control modification steps. (**A**) Tests using pig serum that tested negative for PCV-2. AuNPs (black line), MUA (red line), EDC–NHS (green line), antibody 2.5 µg/mL (blue line), and pig serum negative for PCV-2 (pink line). (**B**) Potential interferences analysis. AuNPs (black line), MUA (red line), EDC–NHS (green line), antibody 2.5 µg/mL (blue line), adenovirus (yellow line), parvovirus (pink line), PCV-1 (light blue line), and PCV-2 (orange line).

Tests using pig serum that tested negative for PCV-2 were performed based on the qPCR to evaluate the experimental specificity and selectivity (Figure 6). Five samples from different animals were tested in triplicate, and their results are shown in Figure 6A. AuNPs without surface modification showed an absorbance peak of 1.44 at 526 nm (black line). SAMs formation resulted in an absorbance peak decrease to 1.42 (red line). EDC–NHS solution addition resulted in an absorbance peak decrease to 1.33 and in a wavelength shift to 529 nm (green line). Antibody solution (2.5 µg/mL) addition resulted in an absorbance peak decrease to 1.16 and in a wavelength shift to 532 nm (blue line) if one takes into consideration antibody binding to the AuNPs surface. After that, a PCV-2 negative serum sample was added (pink line). This outcome indicated that there was not enough strong antigen–antibody binding to change the resonance of the electrons found on the AuNPs surface.

In addition, we evaluated the detection of viruses in the presence of potential interferences to validate the proposed methodology. PCV-2 is a non-enveloped DNA virus belonging to the family *Circoviridae.* Non-enveloped DNA viruses belonging to the *Parvoviridae* and *Adenoviridae* families were tested to verify the binding specificity of the antibody used [35,36]. Another virus tested was the PCV-1, belonging to the same family of PCV-2 presenting similar structural proteins, which can cause false positive diagnoses in conventional tests (Figure 6B). The UV-Vis graph shows the formation of the complex. AuNPs (black line), AuNPs/MUA (red line), AuNPs/MUA/EDC–NHS (green line), and AuNPs/MUA/EDC–NHS/antibodies 2.5 µg/mL (blue line) had an absorbance peak of 0.79 at 547 nm. After addition of the adenovirus sample (yellow line), the absorbance peak was 1.07 at 534 nm. Parvovirus when added to the complex showed a peak absorbance of 0.78 at 545 nm (pink line) and the PCV-1 absorbance peak was 0.86 at 545 nm (light blue line). For positive control and easy comparison of the displacement peak in the graph, a PCV-2 sample was added to the complex and showed an absorbance peak of 0.30 at 608 nm (orange line). Comparing the PCV-2 peak with the interferences’ analyses, we observed that there was no decrease in the absorbance peak and wavelength shift, which was characterized by no increase in the local refractive index, showing that only the PCV-2 bound to the AuNPs–antibody complex.

## 4. Conclusions

The current study introduced a methodology for PCV-2 diagnosing in pig serological samples. The conjugation between AuNPs and antibodies by SAMs has formed a stable and specific complex for the PCV-2, which kept the reproducibility of the results. The methodology also proved to be simple and easy to be implemented in comparison to traditionally techniques such as qPCR. The final cost of the analysis was also significantly lower: $3 per sample. Since the disease is caused by an immun-suppressive agent, animals become more prone to develop other diseases, as well as to present emaciation, which has further negative impact on farmers’ incomes. Early PCV-2 detection allows applying the proper treatment to pigs.
